# Severe Thrombocytopenia Associated with Dengue Fever: An Evidence-Based Approach to Management of Thrombocytopenia

**DOI:** 10.1155/2022/3358325

**Published:** 2022-08-12

**Authors:** Sulagna Das, Charles Abreu, Micah Harris, John Shrader, Satish Sarvepalli

**Affiliations:** ^1^Department of Medicine, Kettering Health, Dayton, OH, USA; ^2^Boonshoft School of Medicine, Wright State University, Dayton, OH, USA

## Abstract

Dengue is a mosquito-borne viral illness common in tropical and subtropical countries but very rare in the United States. Patients infected with dengue often present with thrombocytopenia. In the setting of dengue, platelet transfusions as a treatment for thrombocytopenia have no clear benefits in reduction of severe bleeding or improvement of the platelet count. Here, we present a case of a traveler infected with dengue virus and discuss the approach to treat thrombocytopenia.

## 1. Introduction

The dengue virus (DENV) is the most common arbovirus in the world transmitted by the Aedes aegypti mosquito. The virus belongs to the Flaviviridae family, and the disease can be spread by one of the four types: DENV-1, DENV-2, DENV-3, and DENV-4. Dengue is rare in the United States and is often described as the “fever in the returned traveler” due to its common presentation in travelers returning from an endemic region such as the tropical areas of Southeast Asia and the Caribbean [1, 2].

The course of illness ranges from asymptomatic to life-threatening, and incubation typically lasts three to fourteen days. Secondary infection with a second serotype often results in a worse prognosis. The three phases of infection include febrile, critical, and recovery. The febrile phase begins around day four to seven of infection and lasts around three to seven days. Fever is sudden during onset and typically high (greater than 40°C), lasting two to seven days. It can be accompanied by myalgias, arthralgias, nausea, vomiting, diarrhea, abdominal pain, lymphadenopathy, hepatomegaly, maculopapular rash, leukopenia, thrombocytopenia, and transaminitis [3]. The critical phase of infection can be followed after defervescence and is more common in patients with secondary infection and other comorbidities [4]. Thrombocytopenia is common and can occur with vascular leakage, shock, and organ impairment [5]. Theorized mechanisms for thrombocytopenia include platelet activation, which causes attachment to the vascular wall forming thrombi and removal from circulation, immune cytotoxic effects through opsonization of platelets and direct infection of platelets, and megakaryocytes by the virus [6]. The recovery phase occurs with resolution and stabilization of vitals. Diagnosis is with serology of antidengue IgM, and treatment is supportive [3].

Given the rarity of dengue in the United States, physicians should start to consider dengue when patients present with a history of travel from an endemic region and suggestive clinical symptoms. Here, we describe a case of dengue fever in a traveler from an endemic region presenting with severe thrombocytopenia.

## 2. Case

**Figure 1:**
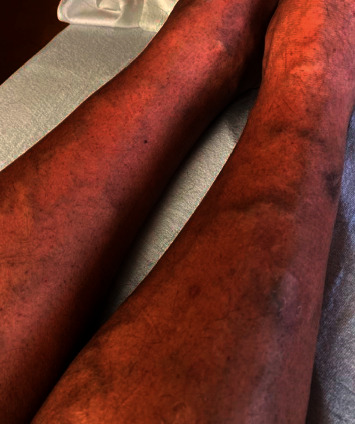
Petechiae.

A 47-year-old Asian Indian male with no significant past medical history, who came from India four days prior to admission, presented with fever with diffuse body aches to hospital in the Midwestern United States. He stated that he was fully vaccinated for COVID-19. As stagnant water is a breeding source for mosquitoes when asked, the patient did mention having air conditioning (AC) in his house, a common source for stagnant water. He stated that he turned his AC on a few times this year, and then, for the last two months, it had been off. The patient's mother was diagnosed with dengue recently in India. The patient also reported that since it is very common to get mosquito bites in India, he did not pay attention and was not sure if he recently noticed increased mosquito bites. The patient denied any petechiae, hematuria, hematemesis, or melena. On admission, his labs were significant for creatine kinase 999 U/L, lactate dehydrogenase 663 U/L, platelet count 41 K/*μ*L, D-dimer 2664 ng/mL, and aspartate aminotransferase (AST) 141 U/L. Peripheral smear was negative for malaria, and Lyme serology was negative. On day 2 of admission, the platelet count dropped to 11 K/*μ*L, and he received 1 unit of platelet transfusion with improvement of his platelet count to 18 K/*μ*L, but within the next 6 hours, the platelet count dropped to 10 K/*μ*L. Infectious disease and hematology-oncology were consulted, and the recommendation was given to not transfuse platelets anymore unless platelets dropped below 10 K/*μ*L as it could increase the risk of volume overload. On day three, the patient was noted to have petechiae on his lower extremities (Figure 1). Over the next few days, the platelet counts continued to fluctuate between 11 K/*μ*L and 18 K/*μ*L. From day five, continued improvement was noted in the platelet count, and the creatinine kinase level came down to reference range levels. Petechiae resolved by day seven, and the patient's appetite continued to improve. The patient was discharged on day seven when his platelet was 35 K/*μ*L, and he had no episodes of bleeding. On outpatient follow-up two days later, his platelet count had improved to 172 K/*μ*L. Two weeks later, his dengue fever antibody IgG resulted positive with a titer of 7.07 ISR.

## 3. Discussion

Thrombocytopenia is a very common clinical manifestation in dengue. Although there are several hypotheses, the mechanisms involved in thrombocytopenia and bleeding manifestation during DENV infection are not fully understood. By interrupting their function, DENV could directly or indirectly affect bone marrow progenitor cells [7] to reduce the proliferative capacity of hematopoietic cells [8]. Evidence suggests that DENV can induce bone marrow hypoplasia during the acute phase of the disease [9]. Thrombocytopenia in dengue may arise either from decreased production of cells from the bone marrow or from increased peripheral destruction of platelets and clearance from peripheral blood. A high mean platelet volume (MPV) indicates enhanced platelet destruction in patients. MPV is usually either high or normal in dengue patients; therefore, excessive platelet destruction could be the main reason for thrombocytopenia in dengue patients.

The cross-reactive antibodies anti-NS1, prM, and E viral proteins against platelets, endothelial cells, or coagulatory molecules may cause platelet dysfunction, endothelial cell damage, coagulation defects, and macrophage activation. Hemorrhage and plasma leakage in dengue hemorrhagic fever result from vascular fragility due to impaired platelet functions. Several studies showed that the platelet count in dengue hemorrhagic fever (DHF)/dengue fever showed a significant decrease on the fourth day of the illness. In fact, previous studies reported DHF in adults without shock, in which platelet counts mildly to moderately decreased on the third day until the seventh day of illness and reached normal levels on the eighth or ninth day [10–12]. In our case, the platelet count started to improve from day seven and came to normal by day ten.

According to clinical guidelines, platelet transfusions should be given to patients who develop serious hemorrhagic manifestations or have very low platelet counts, platelet counts falling below 10–20 × 10^9^/L. In a study of 106 pediatric patients with dengue with thrombocytopenia and coagulopathy, there was no significant difference in hemorrhage between patients who received preventive transfusions compared to those who did not. Patients who received transfusion had a higher frequency of pulmonary edema and the increased length of hospitalization [13]. There were significant side effects with platelet transfusions, and it did not prevent the development of severe bleeding or shorten the time to bleeding cessation. Thus, according to the authors, platelet transfusions should not be routinely performed in the management of dengue [14, 15].

## 4. Conclusion

Since dengue viral illness is quite rare in the United States, clinicians should always consider dengue in their differentials if travelers from tropical countries present with suggestive symptoms. Thrombocytopenia is a very common clinical manifestation in dengue. The platelet count may drop very abruptly, but most of the time it follows its own clinical course and should be managed conservatively. It is very important for clinicians to know that platelet transfusions have no benefit in treating thrombocytopenia in dengue and should be avoided as they may cause significant side effects.
